# First Attempt at Using Electrical Impedance Tomography to Predict High Flow Nasal Cannula Therapy Outcomes at an Early Phase

**DOI:** 10.3389/fmed.2021.737810

**Published:** 2021-10-08

**Authors:** Zhe Li, Zhiyun Zhang, Qian Xia, Danling Xu, Shaojie Qin, Meng Dai, Feng Fu, Yuan Gao, Zhanqi Zhao

**Affiliations:** ^1^Department of Critical Care Medicine, Renji Hospital, School of Medicine, Shanghai Jiao Tong University, Shanghai, China; ^2^Department of Biomedical Engineering, Fourth Military Medical University, Xi'an, China; ^3^Institute of Technical Medicine, Furtwangen University, Villingen-Schwenningen, Germany

**Keywords:** high flow nasal cannula, electrical impedance tomography, acute respiratory failure, ventilation distribution, outcome prediction

## Abstract

**Objective:** Spatial and temporal ventilation distributions in patients with acute respiratory failure during high flow nasal cannula (HFNC) therapy were previously studied with electrical impedance tomography (EIT). The aim of the study was to explore the possibility of predicting HFNC failure based on various EIT-derived parameters.

**Methods:** High flow nasal cannula failure was defined reintubation within 48 h after HFNC. EIT was performed with the patients spontaneously breathing in the supine position at the start of HFNC. EIT-based indices (comprising the global inhomogeneity index, center of ventilation, ventilation delay, rapid shallow breathing index, minute volume, and inspiration to expiration time) were explored and evaluated at three time points (prior to HFNC, T1; 30 min after HFNC started, T2; and 1 h after, T3).

**Results:** A total of 46 subjects were included in the final analysis. Eleven subjects had failed HFNC. The time to failure was 27.8 ± 12.4 h. The ROX index (defined as SpO_2_/FiO_2_/respiratory rate) for HFNC success patients was 8.3 ± 2.7 and for HFNC failure patients, 6.2 ± 1.8 (*p* = 0.23). None of the investigated EIT-based parameters showed significant differences between subjects with HFNC failure and success. Further subgroup analysis indicated that a significant difference in ventilation inhomogeneity was found between ARDS and non-ARDS [0.54 (0.37) vs. 0.46 (0.28) as evaluated with *GI, p* < 0.01]. Ventilation homogeneity significantly improved in ARDS after 60-min HFNC treatment [0.59 (0.20) vs 0.57 (0.19), T1 vs. T3, *p* < 0.05].

**Conclusion:** Spatial and temporal ventilation distributions were slightly but insignificantly different between the HFNC success and failure groups. HFNC failure could not be predicted by changes in EIT temporal and spatial indexes of ventilation distribution within the first hour. Further studies are required to predict the outcomes of HFNC.

## Introduction

High flow nasal cannula therapy may help avoid invasive mechanical ventilation in patients with hypoxemia (1). Recent studies have indicated that a high flow nasal cannula (HFNC) improves respiratory drive and lung mechanics and enhances CO_2_ removal (2, 3). HFNC introduces low levels of airway pressure, which increase end-expiratory lung volume and improve oxygenation and regional lung aeration (4). However, in cases of HFNC failure, a delay in mechanical ventilation may result in deterioration in patient outcomes. Therefore, early prediction of HFNC outcomes is important in order for clinicians to decide the need for mechanical ventilation. Higher simplified acute physiology score II, the severity of hypoxemia, and C-reactive protein level may be correlated with HFNC failure (5, 6). A simple index calculated as the ratio of SpO_2_/FiO_2_ to respiratory rate may be able to identify HFNC failure after a 12-h trial (7). This index does not directly reflect ventilation status and may, therefore, require a longer period to identify the HFNC failure.

Electrical impedance tomography (EIT) is a novel noninvasive, radiation-free, bedside method for monitoring ventilation changes related to different lung conditions. These include lung regional recruitment and overdistension during positive end-expiratory pressure (PEEP) titration in patients with acute respiratory distress syndrome (8–11). Various EIT-based parameters were proposed to evaluate the status of ventilation in patients with spontaneous breathing (12, 13). In addition, previous studies have shown that EIT can be used to assess the effect of HFNC on regional ventilation (14, 15).

This study aimed to describe the evolution of spatial and temporal ventilation distributions in patients with acute respiratory failure (ARF) during the first hour of HFNC. Furthermore, we examined the possibility of predicting HFNC failure based on regional ventilation information derived from the EIT.

## Methods

### Subjects and Measurement

The study protocol was approved by the ethics committees of Renji Hospital, School of Medicine, Shanghai Jiao Tong University (KY2021-057-B). Written informed consent was obtained from all the subjects before the study.

Patients who were treated with HFNC after ICU admission from 2021.05.27 to 2021.06.20 were screened. Only patients with acute respiratory failure ARF (respiratory rate >25 breaths/min, PaO_2_/FiO_2_ < 300 mmHg) were included. Exclusion criteria included age <18 years, pregnancy, and lactation period, weaning from the ventilator, intubation required, tracheotomy, bronchoscopy, absence of commitment to pursue full life support, and any contraindication to the use of EIT (pacemaker, automatic implantable cardioverter defibrillator, and implantable pumps).

High flow nasal cannula was performed with Optiflow (Fisher and Paykel Healthcare, East Tamaki, New Zealand) or HFNC module in V300 or V500 (Dräger Medical, Lübeck, Germany). The initial flow setting was 50–60 L/min with heated and humidified oxygen (FiO_2_ = 1). When peripheral oxygen saturation was over 92%, FiO_2_ was reduced gradually. The flow was reduced in the first hour only if the patient felt uncomfortable with the rate. HFNC failure was defined as exacerbation after HFNC, which led to intubation within 48 h. The indications for invasive mechanical ventilation included the level of consciousness (Glasgow coma score <12), cardiac arrest/arrhythmias and severe hemodynamic instability (norepinephrine > 0.1 μg/kg/min), and a persistent or worsening respiratory condition. This was defined as at least two of the following conditions: failure to achieve correct oxygenation (PaO_2_ < 60 mmHg despite HFNC flow ≥ 30 L/min and FiO_2_ of 1), respiratory acidosis (PaCO_2_ > 50 mmHg with pH < 7.25), respiratory rate > 30 bpm or inability to clear secretions. The ROX index [defined as SpO_2_/FiO_2_/respiratory rate (7)] was calculated 1 h after HFNC.

Electrical impedance tomography was performed with the patients spontaneously breathing in the supine position at the start of HFNC. An EIT electrode belt with 16 electrodes was placed around the thorax at the 4th intercostal space, and one reference electrode was placed on the abdomen (PulmoVista 500, Dräger Medical, Lübeck, Germany). Electrical alternating currents were applied in a sequential rotating process. The frequency and the amplitude of the currents were determined automatically according to the background noise of the measurement environment. The resulting surface potential differences between neighboring electrode pairs were measured and recorded at 20 Hz for 1 h. Image reconstruction using this algorithm was performed using the software of the manufacturer (EIT Data Review Tool, Dräger Medical, Lübeck, Germany). The EIT data were analyzed offline with customized software programmed with MATLAB R2015 (The MathWorks Inc., Natick, MA).

### EIT Data Analysis

Functional EIT (fEIT)-tidal variation (TV) was calculated by subtracting the end-expiration from the end-inspiration image, representing the variation during tidal breathing. Tidal images of 1 min were averaged to increase the signal-to-noise ratio.


(1) $$\begin{array}{l} TV_{i}=\frac{1}{N}\overset{N}{\underset{n=1}{\sum }}\left(Z_{i,Ins,n}-Z_{i,Exp,n}\right) \end{array}$$

where *TV*_*i*_ is the pixel *i* in the fEIT image; *N* is the number of breaths within the analyzed period; and Δ*Z*_*i,Ins*_ and Δ*Z*_*i, Exp*_ are the pixel values in the raw EIT image at end inspiration and end expiration, respectively. When *TV*_*i*_ < 0, a value of 0 was assigned to *TV*_*i*_.

Several EIT-based indices were explored and evaluated at three time points (before HFNC, T1; 30 min after HFNC started, T2; 1 h after, T3). They are explained in detail in the remainder of this section. To investigate the changes from the baseline and treatment, the differences of the EIT indices between the time points were calculated and normalized to the values at T1. The normalized values were denoted as Δ_T3−T1_ and Δ_T3−T2_, respectively.

The global inhomogeneity (*GI*) index is calculated from the tidal EIT images to summarize the heterogeneity of ventilation (16).


(2) $$\begin{array}{l} GI=\frac{\sum_{l\in lung}|TV_{l}-Median\left(TV_{lung}\right)|}{\sum_{l\in lung}TV_{l}} \end{array}$$

where *TV* denotes the value of the differential impedance in the tidal images; *TV*_*l*_ is the pixel in the identified lung area; pixel l is considered as a lung region if *TV*_*l*_ > 10% × max (*TV*). *TV*_*lung*_ denotes all pixels representing the lung area. A high-GI index implies high variation among pixel tidal impedance values.

The center of ventilation (*CoV*) depicts the ventilation distribution influenced by gravity or various lung diseases (relative impedance value weighted with a location in the anteroposterior coordinate) (17):


(3) $$\begin{array}{l} CoV=\frac{\sum \left(y_{i}\times TV_{i}\right)}{\sum {TV}_{i}}\times 100\% \end{array}$$

where *TV*_*i*_ is the impedance change in the fEIT image for pixel *i*; *y*_*i*_ is the height of pixel *i*, and the value is scaled such that the bottom of the image (dorsal) is 100% and the top (ventral) is 0%.

The tidal image was divided into four horizontal, anterior-to-posterior segments of equal height (regions of interest, ROI). The ventilation distributions in these regions were calculated and denoted as *ROIs 1-4*. The regional ventilation delay (RVD) index characterizes the regional ventilation delay as pixel impedance rising time compared to the global impedance curve (18), which may be used to assess tidal recruitment/derecruitment.


(4) $$\begin{array}{l} RVD_{l}=\frac{t_{l,40\%}}{T_{inspiration,\;global}}\times 100\% \end{array}$$

where *t*_*l*,40%_ is the time required for pixel *l* to reach 40% of its maximum inspiratory impedance change. *T*_*inspiration, global*_ denotes the inspiration time calculated from the global impedance curve.

Besides the conventional EIT-based indices, we constructed further parameters that are relevant to spontaneously breathing patients but are difficult to record without additional devices. The rapid shallow breathing index (RSBI) is defined as the ratio of the respiratory rate to tidal volume. Since the change in tidal volume can be estimated by the measured impedance, *RSBI*_*EIT*_ was calculated as the ratio of the respiratory rate to tidal impedance variation in arbitrary units. Similarly, minute volume was estimated as the multiplication of the respiratory rate and tidal impedance variation in arbitrary units (*MV*_*EIT*_). Inspiration time over expiration time (*I:E*) was calculated based on the global impedance—time curves.

### Statistical Analysis

Normal distribution was assessed with the Kolmogorov-Smirnov normality test. Normally distributed results were presented as the mean ± SD. Non-normally distributed results were presented as median (interquartile range). The Kruskal–Wallis test was used to compare the parameters at different time points. The Mann–Whitney test was used to compare the EIT parameters between the HFNC success and failure groups. Because EIT can only deliver relative impedance changes, therefore, only the changes in *RVD, RSBI*_*EIT*_, and *MV*_*EIT*_ were compared between groups. A *p* < 0.05 was considered statistically significant. Bonferroni correction was used to adjust the *p*-value for multiple comparisons of different time points or ROIs.

## Results

A total of 48 subjects were included in the study. Two patients were excluded from the final analysis due to poor EIT data quality (intensive patient movement). The demographics and outcomes of the 46 subjects are summarized in Table 1. Totally, 11 patients had failed HFNC. The time to failure was 27.8 ± 12.4 h. The ROX index for HFNC success patients was 8.3 ± 2.7 and for HFNC failure patients, 6.2 ± 1.8 (*p* = 0.23). Twelve patients had a fever (body temperature > 37°C). Two of them were > 38.5°C. Of the HFNC success patients, 21 were not sedated. Of the HFNC failure patients, five were not sedated. No significant differences were found (*p* = 0.40).

**Table 1:** Demographics and outcomes of the studied subjects.

**Pat. No**.	**Age**	**Gender**	**APACHE II**	**Height (cm)**	**Weight (kg)**	**init**.	**init**.	**ROX**	**HFNC**	**Dead**
						**FiO_**2**_**	**PaO_**2**_**		**success**	
1	53	F	10	160	56	0.90	72	6.67	1	0
2	39	M	8	172	88	0.80	92	8.95	1	0
3	77	F	12	159	57	0.55	97	5.00	1	0
4	74	M	8	175	68	0.55	78	8.82	0	1
5	82	M	10	177	68	0.80	87	5.94	1	0
6	89	M	16	174	65	0.40	56	6.96	1	0
7	82	M	18	170	68	0.55	68	12.82	1	0
8	67	M	14	173	69	0.70	45	8.67	1	0
9	50	M	8	168	59	0.80	69	7.68	1	0
10	67	M	24	173	68	0.55	82	9.90	1	0
11	35	M	25	170	85	1.00	184	4.44	1	0
12	67	M	20	172	69	0.50	69	5.35	0	0
13	75	M	15	176	64	0.50	47	4.69	0	0
14	72	M	22	178	63	0.60	73	6.94	0	1
15	65	F	15	158	56	1.00	84	4.78	0	0
16	79	M	18	171	59	1.00	64	6.65	0	1
17	76	F	24	158	63	0.60	73	3.07	0	1
18	52	M	24	172	70	0.55	70	6.40	1	0
19	66	M	13	174	69	0.60	51	5.22	1	0
20	72	M	18	175	73	0.60	84	9.26	1	0
21	66	M	21	173	69	0.40	68	4.85	0	0
22	77	M	24	178	65	0.45	61	7.84	1	0
23	83	M	18	169	82	0.80	232	6.94	1	0
24	69	M	24	173	69	1.00	58	6.67	1	0
25	62	M	17	176	68	0.50	65	6.74	1	0
26	66	M	16	174	71	0.50	130	9.26	1	0
27	72	M	18	172	61	0.45	68	8.25	1	0
28	68	M	16	170	58	0.50	100	7.25	1	1
29	77	F	12	155	49	0.50	67	6.27	1	0
30	73	M	17	172	79	0.55	63	6.17	0	1
31	77	M	25	173	72	0.55	83	5.29	1	0
32	51	F	24	155	55	0.60	84	8.98	1	0
33	57	M	25	168	58	0.60	68	7.84	1	0
34	66	F	18	155	57	0.50	65	8.51	1	0
35	66	M	14	173	65	0.50	82	11.70	1	0
36	63	M	17	174	68	0.50	67	8.17	0	1
37	31	F	20	161	56	0.40	57	6.11	1	0
38	54	M	14	175	68	0.50	70	18.46	1	0
39	63	F	13	156	63	0.33	89	10.00	1	0
40	52	M	14	172	65	0.70	153	7.57	1	0
41	82	M	24	172	55	0.50	60	11.53	1	0
42	67	F	17	158	65	0.50	69	8.61	0	0
43	74	M	18	178	59	0.50	62	11.00	1	0
44	52	M	24	169	64	0.50	65	9.51	1	0
45	73	M	24	174	59	0.90	50	8.08	1	0
46	58	F	24	158	51	0.70	83	10.42	1	0
Mean	66	M:F	18	169	65	0.61	80	7.83	s:f	d:a
SD	13	35:11	5	7	8	0.18	34	2.65	35:11	7:39

*Pat. No., patient number; SD, standard deviation; M, male; F, female; APACHE II, Acute Physiology and Chronic Health Evaluation II; init, initial; FiO_2_, fractional inspired oxygen; PaO_2_, arterial oxygen partial pressure; s:f, success vs. failure; d:a, dead vs. alive*.

*FiO_2_ and PaO_2_ are values recorded during oxygen therapy before high flow nasal cannula (HFNC)*.

*ROX index, SpO_2_/FiO_2_/respiratory rate HFNC success was marked 1 and failure marked 0*.

*Dead marked subjects died in the ICU*.

Table 2 summarizes the main causes of ARF and the comorbidities of the study subjects. ARF was mainly caused by acute respiratory distress syndrome (ARDS) and pneumonia. Hypertension was the most common comorbidity.

**Table 2:** Summary of primary etiology for ARF and the comorbidities.

	**HFNC success**	**HFNC failure**
*n* = 46	35	11
**Primary etiology for ARF**		
ARDS	15	5
Pneumonia	12	5
Cardiogenic pulmonary edema	7	0
Pulmonary embolism	1	1
**Comorbidity**		
Hypertension	12	7
Congestive heart failure	5	4
Cerebral vascular disease	4	2
Chronic kidney disease	4	3
Chronic pulmonary disease	4	0
Diabetes	7	4

*ARF, acute respiratory failure; ARDS, acute respiratory distress syndrome*.

**Figure 1:**
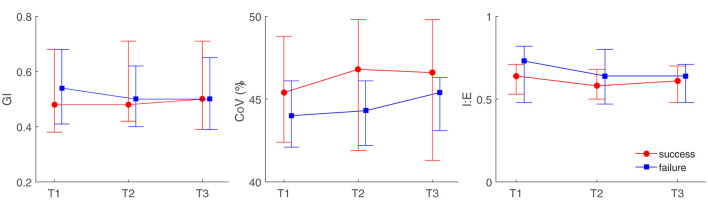
Illustration of electrical impedance tomography (EIT)-derived parameters at different time points between high flow nasal cannula (HFNC) success and failure groups. GI, the global inhomogeneity index; CoV, center of ventilation; I:E, inspiration to the expiration time. T1, before HFNC; T2, 30 min after HFNC started; T3, 1 h after HFNC started.

**Figure 2:**
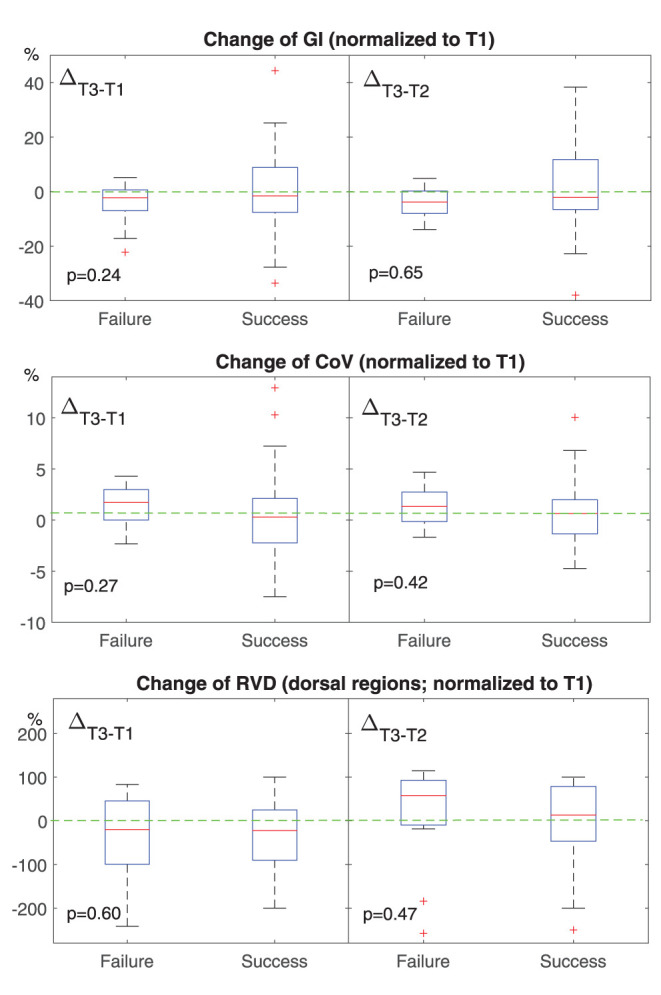
Change of conventional EIT-based parameters at different time points. T1, before HFNC; T2, 30 min after HFNC started; T3, 1 h after HFNC started. All values were normalized to that at T1. The boxes mark the quartiles with median marked red, while the whiskers extend from the box out to the most extreme data value within 1.5^*^ the interquartile range of the sample. The red crosses are outliers. Green dashed lines marked the value of 0. GI, the global inhomogeneity index. Lower than 0 means the ventilation becomes more homogeneous. CoV, the center of ventilation. Higher than 0 means that the ventilation distribution moves toward dorsal regions. RVD, regional ventilation delay. Higher than 0 means the delay is getting worse.

**Figure 3:**
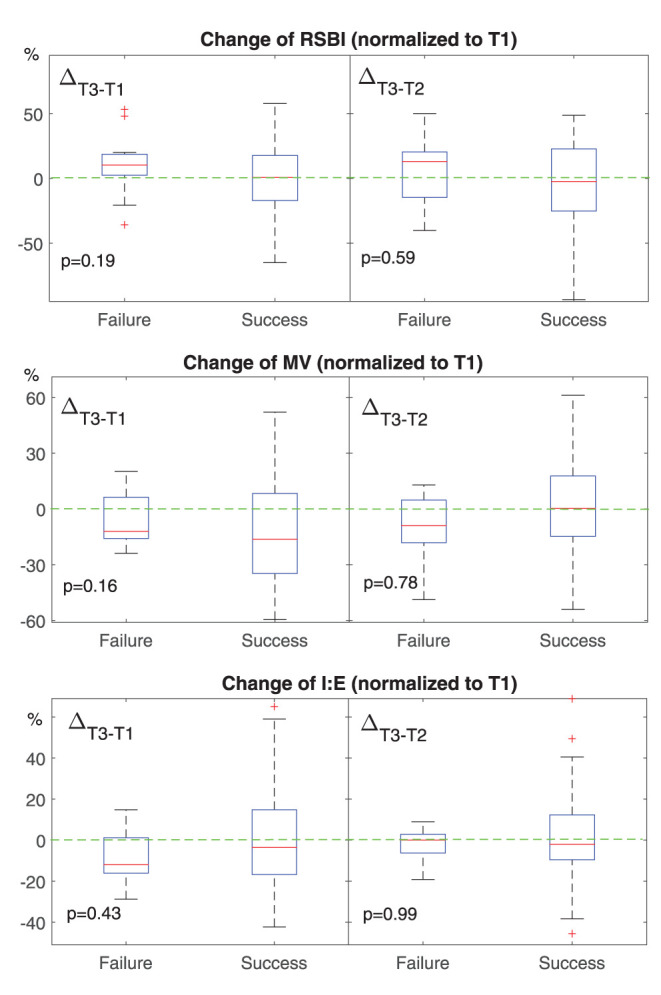
Change of EIT-based parameters for spontaneous breathing at different time points. T1, before HFNC; T2, 30 min after HFNC started; T3, 1 h after HFNC started. All values were normalized to that at T1. The boxes mark the quartiles with median marked red, while the whiskers extend from the box out to the most extreme data value within 1.5^*^ the interquartile range of the sample. The red crosses are outliers. Green dashed lines marked the value of 0. RSBI, rapid shallow breathing index. Higher than 0 means the subject is breathing more rapidly or shallowly. MV, minute volume. Lower than 0 means the minute volume becomes less at T3. I:E, inspiration to the expiration time. Lower than 0 means the inspiration time is getting shorter.

None of the investigated EIT-based parameters showed significant differences between the subjects with HFNC failure and success. Table 3 summarizes the absolute values of the EIT-derived parameters at different time points (*GI, CoV*, and *I:E*). Figure 1 shows the trends of EIT parameters at different time points. Figures 2, 3 compare the EIT-based parameters at different time points between the groups with HFNC failure and success. In the failure group, ventilation was distributed slightly toward the dorsal regions, as indicated by *CoV* (Figure 2, middle; *p* > 0.05). Ventilation delay decreased in both failure and success groups compared to T1, but, as compared to T2, the ventilation delay was worse at T3 in the failure group (Figure 2, bottom; *p* > 0.05). Elevated *RSBI*_*EIT*_ and shorter inspiration time (lower *I:E*) were found in the failure group at T3 compared to T1 (Figure 3, top; *p* > 0.05). *MV*_*EIT*_ decreased in the HFNC failure group compared to that in T2, while the median in the success group was higher (Figure 3 middle; *p* > 0.05).

**Table 3:** A summary of the absolute values of electrical impedance tomography (EIT)-derived parameters and the comparison between groups at different time points.

		**HFNC success**	**HFNC falure**	***p***
GI	T1	0.48 (0.38–0.68)	0.54 (0.41-0.68)	0.63
	T2	0.48 (0.42–0.71)	0.50 (0.40–0.62)	0.78
	T3	0.50 (0.39–0.71)	0.50 (0.39–0.65)	0.94
CoV (%)	T1	45.4 (42.4–48.8)	44.0 (42.1–46.1)	0.61
	T2	46.8 (41.9–49.8)	44.3 (42.2–46.1)	0.39
	T3	46.6 (41.3–49.8)	45.4 (43.1–46.3)	0.72
I:E	T1	0.64 (0.53–0.71)	0.73 (0.48–0.82)	0.36
	T2	0.58 (0.5–0.68)	0.64 (0.47–0.80)	0.61
	T3	0.61 (0.48–0.70)	0.64 (0.48–0.71)	0.88

Further subgroup analysis indicated that significant difference in ventilation inhomogeneity was found between ARDS and non-ARDS [0.54 (0.37) vs. 0.46 (0.28) as evaluated with *GI, p* < 0.01]. Ventilation homogeneity significantly improved in ARDS after 60 min HFNC treatment [0.59 (0.20) vs 0.57 (0.19), T1 vs. T3, *p* < 0.05].

## Discussion

In the present study, we have examined the ability to predict the outcome of HFNC within the first hour of treatment using EIT. Differences in spatial and temporal ventilation between the HFNC failure and success groups were observed, but they were not statistically significant.

Previous studies indicated that HFNC failure may lead to intubation delay and increase hospitalization time and mortality. Roca et al. showed that the HFNC failure rate in patients with severe pneumonia was up to 28% (19). Rello et al. found that, in patients with confirmed 2009 influenza A/H1N1v infection, the mortality in the HFNC failure group was 27%, whereas the mortality in initially intubated patients was only 20% (20). Kang et al. revealed in a retrospective study that the mortality was significantly higher in patients intubated after 48-h HFNC failure compared with those who were intubated within 48-h HFNC (21). The extubation rate was lower in the HFNC failure after 48 h. Recent studies have indicated that the mortality rate in intubated patients after HFNC failure was around 30–50% (22). When the respiratory drive of the patient was too high, the high-flow rate might induce overdistension (23). EIT has been used to monitor the ventilation during HFNC. A recent study has indicated that EIT can help to identify the overdistension caused by HFNC (15). Besides, EIT can observe pendelluft and diaphragm activities and monitor the corresponding lung injury, which may help to identify the respiratory drive of the patient (24–26).

In previous studies, EIT has been used mainly as a monitoring tool to demonstrate its superiority over other ventilation modes. For example, HFNC was found to be superior to conventional oxygen therapy or noninvasive ventilation in regard ventilation distribution, end-expiratory lung volume, and respiratory rate, etc., (27, 28). However, the ability to predict the HFNC outcomes has yet to be explored. Previously, the so-called ROX index was proposed (19). The ROX index is relatively easy to obtain and has great potential to predict HFNC outcomes within a few hours after initiating HFNC (7, 19). However, it seems that its effective cutoff value varies depending on the time point of observation and disease. For our study patients (mainly lung healthy subjects after abdominal surgery), the specificity of ROX at 1 h was low (0.36), which is not enough to identify HFNC failure. On the other hand, HFNC results in ventilation redistribution within a brief period as demonstrated in previous studies [e.g., (4, 15)]. Therefore, we took the challenge, attempting to predict the outcome of HFNC within the first hour of treatment. For the selection of EIT-based parameters, we have tested some of the most widely used EIT indices, such as *GI* and *CoV* (29). In addition, the equivalent parameters *RSBI*_*EIT*_, *MV*_*EIT*_, and *I:E* were evaluated because the ventilated volume was not usefully monitored during HFNC.

We found that ventilation was distributed slightly toward the dorsal regions in the failure group (Figures 1, 2, *CoV*). Previous studies suggested that ventilation distribution in the dorsal regions might be associated with diaphragm activity (25). The differences found in *CoV* might indicate an increased respiratory effort in the failure group, which implied an unsatisfactory oxygen delivery. Due to the nature of the study (observational design), we did not include the measurements of transdiaphragmatic pressure or diaphragmatic ultrasound to confirm this speculation. A similar trend of *RSBI*_*EIT*_ was observed that might as well support our hypothesis (Figure 3, *RSBI*). When the respiratory muscle was fatigued, the inspiration time became shorter and MV decreased at T3 compared to the baseline (Figure 3). Ventilation delay at T3 decreased in both failure and success groups compared to T1, but, as compared to T2, it deteriorated in the failure group (Figure 2 *RVD*). The *RVD* index was initially developed and evaluated during low-flow maneuvers (16). We suspect that, during spontaneous breathing, the inspiration time is too short to have a stable *RVD* value. A recent study has shown that the coefficient of variation for *RVD* in healthy subjects was high (30), which might be the reason why no significant difference could be found in the present study.

Another potential parameter that could be used to evaluate the effect of HFNC is the change in end-expiratory lung impedance (ΔEELI), which is associated with end-expiratory lung volume. Mauri et al. evaluated the lung mechanics during HFNC in 17 patients with respiratory failure (2). They found that ΔEELI increased and MV decreased with an increasing flow rate during HFNC. We did not explore the parameter ΔEELI for two reasons: (1) As suggested by a previous study (15), HFNC may introduce overdistension as well. Overdistension would lead to an increased EELI but decreased tidal volume, which may explain the finding in the study of Mauri (2). (2) In our study, the impedance value was not normalized to volume so that the ΔEELI was not inter-patient comparable.

Unfortunately, none of the explored EIT-based parameters showed statistical significance when comparing the HFNC success and failure groups. We suspected that a large portion of the included subjects was admitted to ICU after abdominal surgery. Their lung function might be satisfactory, but the pain from the wound might have influenced their respiratory muscles. Moreover, the sample size was small, and only a few patients required intubation. Since this was the first attempt to use EIT to predict an HFNC outcome, no *a priori* information was available to calculate the sample size. The study could be underpowered depending on which parameter was being evaluated. Further studies can be designed based on the current findings. The subject group must be carefully selected. Another limitation of the study design was that only the first hour of EIT data was recorded so that the observation period was very short. It is unclear whether the changes in spatial and temporal ventilation distribution at a later time point could predict the HFNC outcomes.

## Conclusion

Spatial and temporal ventilation distributions were slightly but insignificantly different for HFNC success and failure groups. HFNC failure could not be predicted by changes in EIT temporal and spatial indexes of ventilation distribution within the first hour. Further studies are required to develop an early indicator to predict the outcome of HFNC.

## Data Availability Statement

The original contributions presented in the study are included in the article/supplementary material, further inquiries can be directed to the corresponding author/s.

## Ethics Statement

The studies involving human participants were reviewed and approved by Ethics Committees of Renji Hospital, School of Medicine, Shanghai Jiao Tong University (KY2021-057-B). The patients/participants provided their written informed consent to participate in this study.

## Author Contributions

ZL, YG, and ZZhao have designed the study. ZZhang, QX, DX, and SQ have performed the measurements and collected the data. ZL, ZZhang, MD, and FF have analyzed the data. ZL, ZZhang, YG, and ZZhao have drafted the manuscript. QX, DX, SQ, MD, and FF have revised the manuscript. All the authors have approved the final version.

## Funding

This study was supported by Shanghai Jiao Tong University (YG2019ZDB04) and the National Natural Science Foundation of China (52077216).

## Conflict of Interest

ZZhao receives a consulting fee from Drager Medical. The remaining authors declare that the research was conducted in the absence of any commercial or financial relationships that could be construed as a potential conflict of interest.

## Publisher's Note

All claims expressed in this article are solely those of the authors and do not necessarily represent those of their affiliated organizations, or those of the publisher, the editors and the reviewers. Any product that may be evaluated in this article, or claim that may be made by its manufacturer, is not guaranteed or endorsed by the publisher.
